# Clinical characteristics and prognosis of pediatric myelin oligodendrocyte glycoprotein antibody-associated diseases in China

**DOI:** 10.1186/s12887-022-03679-3

**Published:** 2022-11-18

**Authors:** Xiaoang Sun, Meiyan Liu, Xiaona Luo, Fang Yuan, Chunmei Wang, Simei Wang, Quanmei Xu, Yuanfeng Zhang, Yucai Chen

**Affiliations:** Department of Neurology, Shanghai Children’s Hospital, School of medicine, Shanghai Jiao Tong University, Shanghai, China

**Keywords:** Myelin oligodendrocyte glycoprotein antibody, Acquired demyelinating syndromes, Clinical features, Prognosis, Children

## Abstract

**Background:**

Research on myelin oligodendrocyte glycoprotein antibody (MOG-Ab)-associated disease (MOGAD) among Chinese children is relatively rare. Therefore, this study aimed to explore and analyze the clinical characteristics and prognoses of Chinese children with acquired demyelinating syndromes (ADSs) who tested positive or negative for MOG-Ab.

**Methods:**

The clinical data of children with MOGAD who were treated in the Department of Neurology at Shanghai Children's Hospital from January 2017 to October 2021 were retrospectively collected.

**Results:**

Among 90 children with ADSs, 30 were MOG-Ab-positive, and 60 were MOG-Ab-negative. MOG-Ab-positive children experienced more prodromal infections than did MOG-Ab-negative children (*P* < 0.05). Acute disseminated encephalomyelitis was the most common ADSs in both groups. There were ten cases of a rebound increase in MOG-Ab titers. There were significant differences in the MOG titer-related prognosis and disease time course between the disease relapse group and the non-relapse group (*P* < 0.01). Among the MOG-Ab-positive patients, the most affected brain areas detected via magnetic resonance imaging (MRI) were the temporal lobe, cerebellar hemispheres, brainstem, and periventricular lesions. The most common shapes of the lesions were commas, triangles, or patches. The average improvement time based on brain MRI was much longer in MOG-Ab-positive than in MOG-Ab-negative children (*P* < 0.05). The initial treatment time correlated with the disease time course, and the prognosis may be affected by the disease time course and serum MOG-Ab titer (*P* < 0.05).

**Conclusion:**

The clinical characteristics and imaging features of ADSs differed between MOG-Ab-positive and MOG-Ab-negative children. In addition to existing treatment plans, additional diagnoses and treatment plans should be developed to reduce recurrence and improve the prognoses of children with MOGAD.

## Background

Acquired demyelinating syndromes (ADSs) are a group of idiopathic immune-mediated diseases that originate in the brain (including the optic nerve) and/or spinal cord and are characterized by myelin damage or loss. The clinical phenotypes of ADSs are diverse, including acute disseminated encephalomyelitis (ADEM), transverse myelitis (TM), optic neuritis (ON), neuromyelitis optica spectrum disorder (NMOSD), and multiple sclerosis (MS). Among ADSs, the clinical and imaging manifestations of some diseases overlap, and it is difficult to differentiate them clinically. Identifying the specific biological markers of each disease with the new generation of antibody detection technologies will play an important role in diagnosing diseases in this spectrum.

Myelin oligodendrocyte glycoprotein (MOG), a demyelinating antibody target, was first discovered 40 years ago [1]. Acquired demyelinating diseases associated with MOG-antibodies (MOG-Abs) are collectively referred to as MOG-Ab-associated disease (MOGAD) [2]. It has been reported hat one-third of children with ADS are MOG-Ab positive [3]. MOGAD has attracted significant academic interest in recent years; however, research involving Chinese children remains rare. In order to gain a deeper understanding of the clinical manifestations, imaging features, and prognoses of MOGAD in Chinese children, the clinical data of MOG-Ab-positive and MOG-Ab-negative children with demyelinating central nervous system (CNS) diseases who were treated in our hospital from January 2017 to October 2021 were retrospectively collected and analyzed in this study, with the aim of improving the understanding of the disease and the ability of doctors to diagnose and treat MOGAD in Chinese children.

## Methods

### Patients’ information

The clinical data of children with ADSs were retrospectively collected from the Department of Neurology of Shanghai Children's Hospital from January 2017 to October 2021. This study was approved by the Ethics Committee of Shanghai Children’s Hospital, and all of the participants’ legal guardians provided written informed consent.

The clinical data collected and recorded included general characteristics (sex, age at disease onset, region, and weight), family history, prodromal manifestations, first episode and main clinical manifestations, laboratory examination results (serum vitamin D level, cerebrospinal fluid [CSF] analysis, brain and spinal cord magnetic resonance imaging [MRI] findings, and autoimmune- and central demyelination-related antibody detection results, among others), physical examination findings, time of appearance of antibody positivity, disease type, treatment plan, disease outcome, and review and follow-up results of antibody titers and MRI assessments.

In terms of geographic location, the southern and northern parts of China are divided by the Qinling Mountains and Huaihe River line. The area to the north of the line is defined as the North, whereas the region to the south of the line is defined as the South. The CNS-related autoantibodies quantified in all patients included the N-methyl-D-aspartate receptor (NMDAR), contactin-associated protein-like 2 (CASPR2), and anti-aquaporin-4 (AQP4) antibodies, and MOG-Ab.

### Grouping

The patients were grouped into MOG-Ab-positive and MOG-Ab-negative groups. The inclusion criteria for the MOG-Ab-positive group were as follows: 1) age ≤ 18 years; 2) clinical manifestations of one or a combination of ADEM, NMOSD, ON, TM, non-ADEM encephalitis, overlap syndrome, and others; 3) serum and/or CSF MOG-Ab positivity; 4) MRI or electrophysiological examination results indicative of CNS demyelination; and 5) complete clinical data availability and the exclusion of other diagnoses. For the MOG-Ab-negative group, the inclusion criteria were as follows: 1) age ≤ 18 years; 2) one or a combination of ADEM, NMOSD, ON, TM, MS, clinically isolated syndrome (CIS), and others; 3) serum and CSF MOG-Ab negativity; 4) MRI or electrophysiological examination results indicative of CNS demyelination; and 5) complete clinical data availability and the exclusion of other diagnoses. The similarities and differences in the clinical characteristics, disease outcomes, and imaging features between the two groups were analyzed.

### Standard diagnostic criteria

The criteria used for classification of the patients were as follows: 1) ADEM, CIS, and MS diagnoses were based on the consensus developed by the initial International Pediatric Multiple Sclerosis Study Group [4]; the diagnostic criteria for NMOSD and other unclassified diseases were based on the consensus outlined in a 2015 study [5]. 2) Encephalitis was defined according to the international criteria for inflammatory or infectious encephalitis [6]; patients who met the criteria for encephalitis but did not fulfill those for ADEM were diagnosed with encephalitis other than ADEM. 3) According to the subtypes of MOGAD, disease recurrence was defined by the European Union (E.U.) pediatric MOG consortium consensus group as a new clinical occurrence, with imaging evidence suggesting that the occurrence was at least one month after the last acute attack. 4) MOG-Ab titer recurrence means that a negative MOG-Ab titer result is followed by another positive result. Repeated MOG-Ab titers include recurrent MOG-Ab titers and persistently elevated titers followed by a decline.

### Antibody titer detection

All pathogenic MOG-immunoglobulin G (MOG-IgG) testing was conducted through cell-based assays (CBA) by the Kingmed Diagnostics Co., Ltd. Human embryonic kidney 293 (HEK293) cells were seeded onto 96-well plates, then co-transfected with a full-length human MOG and plasmid cloning deoxyribonucleic acid 3.1-enhanced green fluorescent protein (pcDNA3.1-EGFP), followed by incubation for another 36 h. Subsequently, the cells were fixed with 4% paraformaldehyde for 20 min before antibody detection.

A specific antibody detection procedure was conducted as follows: first, the corresponding MOG detection kit (containing a MOG cell sheet and a negative control cell sheet) was labeled before rewarming it for 10 min at room temperature. Next, the kit was pre-equilibrated at 37 °C for 30 min, then the sample to be tested is diluted in PBS-10% goat serum according to the gradient (for CSF: stock solution 1:1, 1:10, 1:32, 1:100, 1:320, and 1:1,000 dilutions; for serum: 1:10, 1:32, 1:100, 1:320, 1:1,000, and 1:3,200 dilutions). The patients’ serum or CSF samples were applied to the corresponding cell sheet, incubated at 37 °C for 2 h, then washed four times for 5 min each time. The samples were subsequently incubated with a fluorescent secondary antibody (goat anti-human IgG) for 1.5 h, washed four times for 5 min each time, and finally examined under a microscope.

### Statistical methods

We used Statistical Package for the Social Sciences version 19.0 for data analysis. The measurement data conforming to a normal distribution are expressed as the mean ± standard deviation (SD), and the variables were compared between the two groups using two independent samples t-tests; any measurement data that were not normally distributed are expressed as the median (quartile), and such variables were compared between the two groups using Mann–Whitney test. The Kruskal–Wallis H test was used for comparison between different groups. Enumeration data are expressed by the number of cases (percentage), and chi-squared or Fisher's exact tests were used for comparisons between groups. The Spearman’s rank correlation test was used for correlation analyses. Differences were considered statistically significant at *P* < 0.05, and Bonferroni post hoc corrections were made for multiple comparisons when appropriate.

## Results

### Clinical characteristics

Thirty children with MOG-Ab positivity and 60 children with MOG-Ab negativity were enrolled in the study (Table 1). In both groups of patients, there were more boys than girls, the age at disease onset was six to seven years (means of 6.8 years and 7.2 years, respectively), and most of the patients were from regions of east China, such as Jiangsu, Zhejiang, and Shanghai. Among the MOG-Ab-positive children, ten (33.3%) exhibited symptoms of prodromal infection, compared to three patients (5.0%) in the MOG-Ab-negative group (*P* < 0.05). A minority among both groups had a history of vaccination prior to disease onset; more specifically, two MOG-Ab-positive children were vaccinated prior to disease onset (one received a measles vaccine, and the other received a rabies vaccine). The clinical manifestations of the two groups could be further classified by first symptoms and main symptoms. Encephalopathy was the most common first symptom experienced in both groups (33.2% and 25%, respectively), which manifested as abnormal mental behavior or altered consciousness (depression, lethargy, or restlessness). Visual impairment (30%) was second only to encephalopathy among the first symptoms experienced by the MOG-Ab-positive children. Among the main clinical manifestations, some children exhibited a variety of symptoms that intersected with each other during onset. The most common was disordered movement in both groups (23.3% and 18.3%, respectively), which manifested, for example, as weakness of the one or both lower limbs and unsteady gait. No child in either of the two groups had CIS. ADEM was the most common diagnosis (36.7% and 71.7% in the MOG-Ab-positive and MOG-Ab-negative groups, respectively). In the MOG-Ab-positive group, non-ADEM encephalitis (26.7%) was second in incidence only to ADEM. Among the six cases of ON, the numbers of individuals with unilateral ocular involvement and bilateral involvement were the same. Among the four cases of overlap syndrome, two exhibited NMDAR antibody positivity, and the other two exhibited CASPR2 antibody positivity. There were four cases of MS in the MOG-Ab-negative group, and eight cases of other unclassified diseases. Laboratory examinations indicated that in the two groups, children with inadequate vitamin D levels accounted for the majority (60% and 53.1% respectively), but there was no statistically significant difference between the two groups. The CSF white blood cell counts and protein levels were not significantly higher in the MOG-Ab-positive group (105.96 ± 430.76 × 10^6^/L, 20.18 ± 48.40 mg/L) than in the MOG-Ab-negative group (370.78 ± 475.72 × 10^6^/L, 327.82 ± 183.70 mg/L). In fact, in most children, the oligoclonal-IgG bands (OCBs) could not be detected in the CSF and serum. Particularly, OCBs were detected in only three patients in the MOG-Ab-positive group, of whom two were positive for CSF OCBs, one had the same OCBs in the serum and CSF, and both were transient in a later review.

**Table 1:** Clinical data of children with MOG-Ab-positive and MOG-Ab-negative ADS

	**MOG-Ab-positive (*n* = 30)**	**MOG-Ab-negative (*n* = 60)**	***P*-value**
**Sex (F/M)**	10/20	27/33	0.289
**Age at onset, years**	6.8 ± 4.2	7.2 ± 3.4	0.271
**Weight, kg**	28.94 ± 18.40	29.74 ± 15.72	0.055
**Residence (South/North)**	28/2	60/0	0.206
**Prodromal infection, n (%)**	10 (33.3)	3 (5)	0.001*
**Time of prodromal infection, d**	3 ± 5	0.4 ± 2	0.020*
**Vaccination 1 week before illness onset, n (%)**	2 (6.7)	3 (4.9)	1.000
**First symptom, n (%)**			0.047*
Abnormal mental behavior	3(10.0)	5(8.3)	
Visual impairment	9(30.0)	4(6.7)	
Seizures	3(10.0)	10(16.7)	
Speech disorder	2(6.7)	3(5.0)	
Movement disorder	2(6.7)	14(23.3)	
Involuntary movement	0	2(3.3)	
Altered consciousness	7(23.2)	10(16.7)	
Fever with a headache	2(6.7)	2(3.3)	
Headache and vomiting	2(6.7)	10(16.7)	
**Main symptom, n (%)**			
Abnormal mental behavior	0	1 (1.7)	1.00
Visual impairment	4 (13.3)	3 (5.0)	1.00
Seizures	2 (6.7)	5 (8.3)	0.692
Speech disorder	1 (3.3)	3 (5.0)	1.00
Sensory disturbance	1 (3.3)	0	1.00
Involuntary movement	7 (23.3)	11 (18.3)	0.967
Altered consciousness	1 (3.3)	0	1.00
Sleep disorder	1(3.3)	2 (3.3)	1.00
Neurological dysfunction	2 (6.7)	2 (3.3)	1.00
Fever with a headache	3 (10.0)	5 (8.3)	0.692
Other	0	2 (3.3)	1.00
**Vitamin D level, n (%)**	30	49	0.072
Sufficient	10 (33.3)	10 (20.4)	
Insufficient	2 (6.7)	13 (26.5)	
Deficient	18 (60.0)	26 (53.1)	
**CSF WBC, × 10**^**6**^**/L**	105.96 ± 430.76	20.18 ± 48.40	0.378
**CSF protein, mg/L**	370.78 ± 475.72	327.82 ± 183.70	0.469
**OCB ( +), n (%)**	3 (10)	3 (5)	0.378
**Disease type, n (%)**			< 0.001*
ADEM	11 (36.7)	43 (71.7)	
ON	6 (20.0)	2 (3.3)	
NMOSD	1 (3.3)	3 (5.0)	
Encephalitis	8 (26.7)	0	
Overlap syndrome	4 (13.3)	0	
MS	0	4 (6.7%)	
Other	0	8 (13.3%)	
**Disease duration, d**	170 (83–330.0)	90 (23–366.0)	0.106
**Initial treatment time, d**	13 (8.0–36.0)	21 (10.0–150.0)	0.050*
**Treatment situation**			
Number of times			
IVIG doses	4 ± 2	1 ± 2	< 0.001*
Methylprednisolone pulses	2 ± 1	1 ± 1	0.011*
Number of patients			
IVIG doses	5	10	
Methylprednisolone pulses	24	47	
Rituximab	1	3	
**Disease outcome, n (%)**			0.916
Recovery	25 (83.3)	52 (86.7)	
Relapse	5 (16.7)	8 (13.3)	

Values are expressed as median (range) or count (percentage)

Vitamin D levels (in nmol/L) < 37.5 were classified as deficient, 37.52–74.9 as insufficient, and 75–250 sufficient; no patients had levels > 250

Normal range of CSF white blood cells (WBCs): 0–8 × 10^6^/L; protein: 150–450 mg/L

*MOG-Ab* myelin oligodendrocyte glycoprotein antibody, *CSF* cerebrospinal fluid, *OCB* oligoclonal band, *ADEM* acute disseminated encephalomyelitis, *ON* optic neuritis, *NMOSD* neuromyelitis optica spectrum disorder, *MS* multiple sclerosis, *CIS* clinically isolated syndrome, *IVIG* intravenous immunoglobulin

^*^Statistically significant (*P* < .05)

The two groups of children were followed up for 6–30 months. The treatment time of the MOG-Ab-positive group (mean ± SD: 359 ± 514 d, median: 170 d) was longer than that of the MOG-Ab-negative group (mean ± SD: 304 ± 474 d, median: 90 d); however, the initial treatment time of the MOG-Ab-positive children (mean ± SD: 59 ± 145 d, median: 13 d) was much shorter than that of the MOG-Ab-negative children (mean ± SD: 175 ± 415d, median: 21d) (*P* < 0.05). In the first (acute) stage of the disease, a high-dose methylprednisolone pulse regimen (10–20 mg/kg/d) and/or intravenous immunoglobulin (IVIG, 2 mg/kg) was used for treatment in most patients. Immune therapy was administered when glucocorticoids and IVIG were ineffective. Rituximab was administered to a very small number of children after the above treatments failed. None of the children underwent plasma exchange in our hospital. A significant difference was noted in the number of doses of IVIG administered and hormone shock used between the MOG-Ab-positive children and MOG-Ab-negative children (*P* < 0.05). One MOG-Ab-positive child and three MOG-Ab-negative children were treated with rituximab. The final disease outcomes of the two groups of children tended towards improvement. In the MOG-Ab-positive group, five children relapsed, one of whom initially showed symptoms of ON, such as visual impairment, before showing symptoms of encephalopathy after recurrence. Another patient was initially admitted to hospital because of lethargy symptoms, followed by another admission after 2.5 years because of vision loss.

The MOG-Ab-positive children were divided into two groups according to disease recurrence status for comparative analysis (Table 2). There were significant differences in the MOG-Ab titer-based prognosis (*P* < 0.01). In the MOG-Ab-positive children, the MOG-Ab titers in the relapse group were all recurrent and sustained. The median disease duration was twice that of the non-relapse group (*P* < 0.01), and the average disease relapse time was 774 days. In the MOG-Ab-negative group, the time to disease recurrence was 459 days. In the non-relapse group, the MOG-Ab titers of three children continued to increase initially, and then decreased in subsequent tests.

**Table 2:** Comparison of clinical features of recurrence and non-recurrence in MOG-Ab-positive ADS

	Disease relapse group	Non-disease relapse group		*P*
	5		25	
**Initial treatment time, d**	12 (7–18)		13 (8–44)	0.379
**Age at onset, years**	6 ± 3		7 ± 4	0.584
**MOG-Ab titer prognosis**				0.009*
Non-recurrence	0		17	
Recurrence	5		8	
**Disease duration, d**	300 (248–1658)		150 (53–330)	0.006*

*ADS* acquired demyelinating syndrome, *MOG-Ab* myelin oligodendrocyte glycoprotein antibody

^*^Statistically significant (*P* < .05)

### MOG-Ab titers and prognosis

Among the 30 children with positive serum MOG-Ab titers (Table 3), the titers ranged from 1:10 to 1:1,000, and the serum titers were significantly higher than the CSF titers (ranging from 1:1 to 1:10). In 11 cases (36.7%), the serum MOG-Ab titers became negative after treatment, and the recurrence rate was 33.3%, at 10 cases.

**Table 3:** MOG-Ab titer and prognosis

	Number of cases(%)
**Serum MOG-Ab titer (*n* = 30, range = 1:10‒1000)**	
≤ 1:32	13 (43.3)
≥ 1:100 to < 1:320	10 (33.3)
≥ 1:320	7 (23.3)
**CSF MOG-Ab titer (*n* = 9, range = 1:1‒10)**	
≥ 1:1 to < 1:3.2	3 (37.5)
≥ 1:3.2 to < 1:10	2 (25.0)
≥ 1:10	3 (37.5)
**Serum MOG-Ab titer prognosis**	
Became negative	11 (36.7)
Decreased	4 (13.3)
Maintained	2 (6.7)
Increased, then decreased	3 (10.0)
Became negative, then relapsed	4 (13.3)
Became negative, then relapsed, then became negative	1 (3.3)
Became negative, then relapsed, then decreased	2 (6.7)
Decreased, and then increased	2 (6.7)
Decreased, then increased, then decreased	1 (3.3)

*MOG-Ab* myelin oligodendrocyte glycoprotein antibody, *CSF* cerebrospinal fluid

### Correlation analysis of MOG-Ab titers

**Fig. 1:**
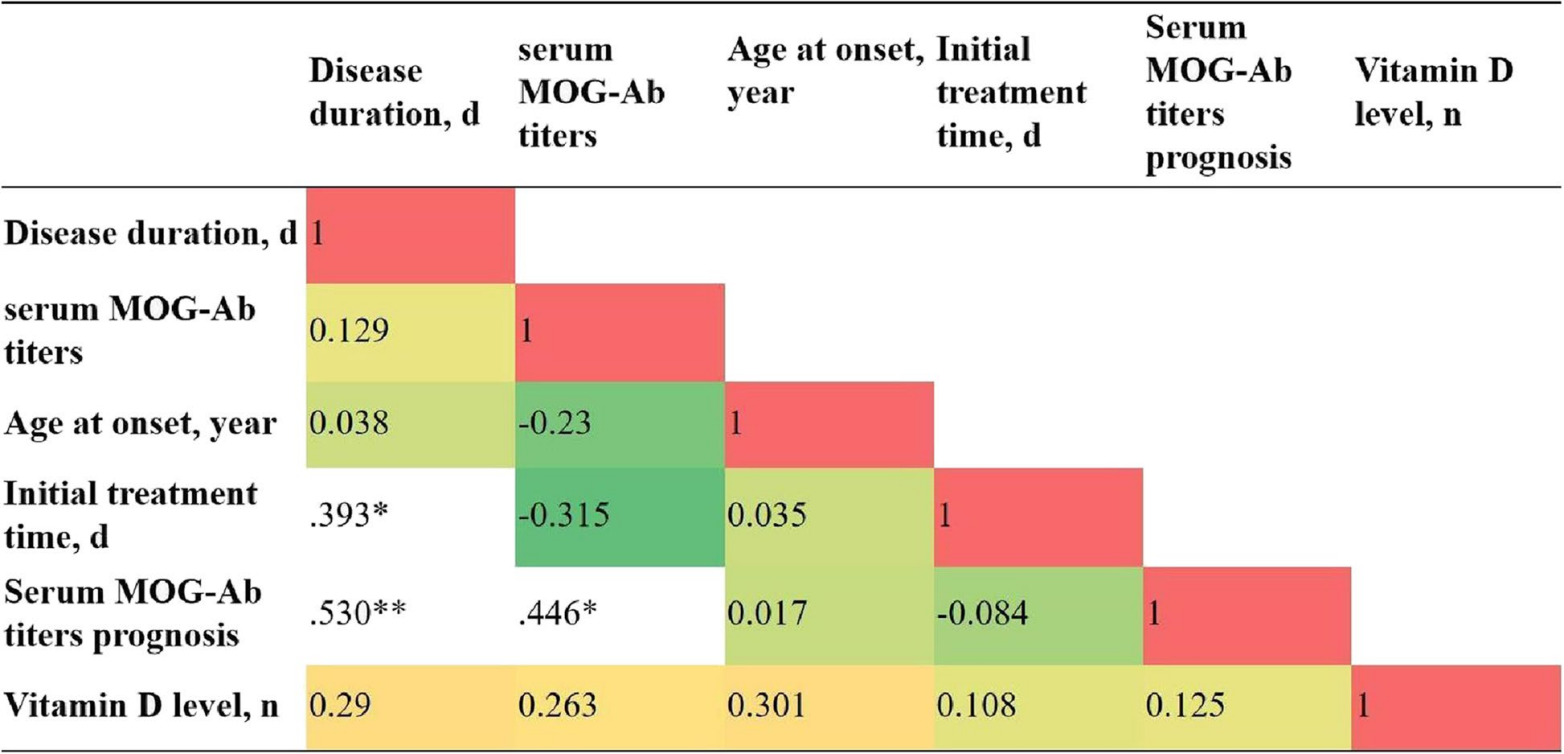
Prognosis correlation analysis of MOG-ab titers *The correlation is significant at a confidence level (two tests) of 0.05; **The correlation is significant at a confidence level (two tests) of 0.01. MOG-Ab, myelin oligodendrocyte glycoprotein antibody

Figure 1 shows that the initial treatment time was positively correlated with the duration of the disease (*P* < 0.05), and the prognosis may be affected by the disease time course and serum MOG-Ab titer (*P* < 0.05 and *P* < 0.01, respectively).

### Imaging features

**Fig. 2:**
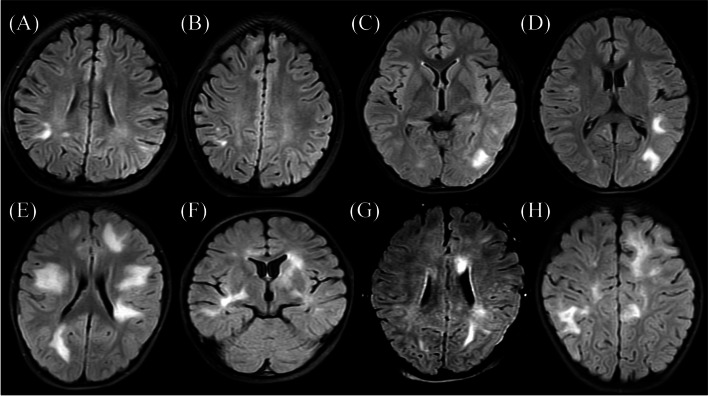
Brain MRI findings of patients with MOG-Ab-positive ADS (**A–H**)

**Fig. 3:**
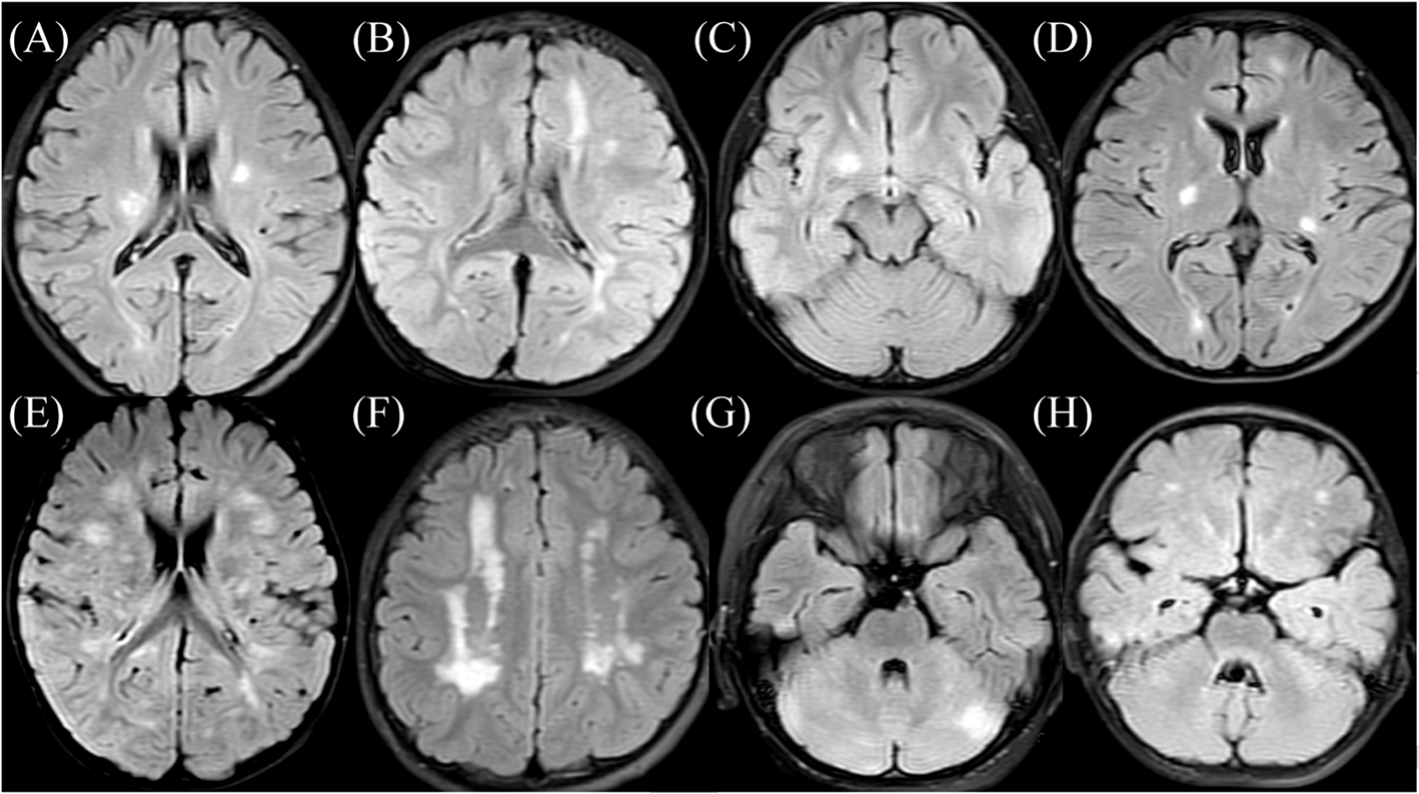
Brain MRI findings of patients with MOG-Ab-negative ADS (**A–H**)

The findings of brain MRI and whole spinal MRI of MOG-Ab-positive and MOG-Ab-negative demyelinating lesions are listed in Table 4. Six MOG-Ab-positive children had normal brain MRI findings. The most common sites of involvement detected via brain MRI in the MOG-Ab-positive children (in eight cases) were the temporal lobe, periventricular lesions, and the cerebellar hemisphere (33.3%); in MOG-Ab-negative children, most cases involved periventricular lesions (26 cases (43.3%). The number of MOG-Ab-positive children with the juxtacortical lesions in the brain was much higher (five times) than that of MOG-Ab-negative children (*P* < 0.05). As shown in Figs. 2 and 3, comparisons of the brain MRI features of MOG-Ab-positive and MOG-Ab-negative children revealed that there were mostly single or multiple abnormal white matter signals in the brain MRI of MOG-Ab-positive children, with clear boundaries, and the common shapes were commas, triangles, or patches. Cervicothoracic involvement was the most common type of spinal cord MRI involvement in both groups. In the MOG-Ab-positive patients, no long segments (lesions involving more than three segments) were found. Follow-up brain MRI, performed at periods ranging from 1 month to 1 year, showed that most patients had small lesions remaining after regular treatment. Moreover, the improvement time based on brain MRI in the MOG-Ab-positive children was nearly two times longer than that of MOG-Ab-negative children (*P* < 0.05).

**Table 4:** Brain and spinal cord MRI involvement and outcomes in MOG-Ab-positive and MOG-Ab-negative ADS

	**MOG-Ab-positive (*n* = 30)**	**MOG-Ab-negative (*n* = 60)**	***P*-value**
	Number of cases	Number of cases	
**Brain involvement site, n (%)**	24	60	
Frontal lobe	6 (25)	19(31.6)	0.546
Parietal lobe	7 (29.2)	18(30.0)	0.940
Occipital lobe	3 (12.5)	8(13.3)	1.000
Temporal lobe	8 (33.3)	8(13.3)	0.035^*^
Basal ganglia	6 (25.0)	16 (26.7)	0.875
Juxtacortical lesions	3 (12.5)	1 (1.67)	0.048^*^
Periventricular lesions	8 (33.3)	26 (43.3)	0.399
Thalamus	7 (29.2)	11 (18.3)	0.274
Cerebellar hemispheres/brainstem	8 (33.3)	15 (25.0)	0.439
**Spinal cord involvement site, n (%)**	30	60	
Cervical	3 (10.0)	7 (11.6)	
Thoracic	2 (6.7)	6 (10.0)	
Lumbar	1 (3.3)	4 (6.7)	
Long segment	0	1 (1.6)	
Conical part	0	1 (1.6)	
**Brain MRI outcome, n (%)**	16	22	0.096
Resolution	4 (25)	2 (9.1)	
Residual small lesions	10 (62.5)	6 (27.3)	
Residual large lesions	0	7 (31.8)	
Progress	2 (12.5)	6 (27.3)	
Progress after improvement	0	1 (4.5)	
**Brain MRI improvement time, d**	60 (27–90)	30 (12–30)	< 0.001^*^

^*^Statistically significant (*P* < .05)

*MOG-Ab* myelin oligodendrocyte glycoprotein antibody, *MRI* magnetic resonance imaging, *ADS* acquired demyelinating syndromes

## Discussion

MOGAD is a rare CNS demyelinating disease that was first recognized only recently. A Dutch study showed that 17% of pediatric patients were MOG-Ab-positive, whereas only 5% of adult patients were MOG-Ab-positive. A national study in Denmark conducted from 2008 to 2018 found that 18% of children with ADS were MOG-Ab-positive [7]. A large study in Austria and Germany found that 31% (65/210) of children with ADS were MOG-Ab-positive [8], and a study in the United Kingdom found that 32% (76/237) of children with ADS were MOG-Ab-positive [9]. Some scholars have also suggested that the incidence of MOGAD in children is more than twice that in adults [10, 11]. The positivity rate of MOG-Ab in our study was 33%, which is slightly higher than that reported in studies among children in other countries. Moreover, the age at disease onset for the MOG-Ab-positive children in our study was approximately 7 years, which is similar to that of other studies [12–14]. There was no obvious trend related to sex in the two groups, although the proportion of males was slightly higher (2:1, 1.2:1, respectively); this finding was slightly different from that of previous studies [15]. Most of our patients were from south China, which may be related to the geographic location of our hospital in the South. Therefore, it is necessary to collect data from multiple centers in different regions for further exploration. In this study, there were six times as many MOG-Ab-positive patients with a history of prodromal infection as MOG-Ab-negative patients, and neurological symptoms occurred within approximately 1 week after fever, which suggests that prodromal infection may play an important role in MOGAD. However, the pathogenesis of MOGAD remains unclear. It is speculated that MOG antigens may leak into the periphery when the permeability of the blood–brain barrier increases (such as in CNS infection) and they are recognized by the immune system. When the blood–brain barrier is damaged again, the MOG-Abs produced in the periphery enter the CNS and cause damage to the nervous system through cytotoxicity mediated by the complement system and antibodies [16].

The spectrum of MOGAD in children is wider than that currently considered in clinical practice, which includes ADEM, NMOSD, ON, encephalitis, and overlap syndrome. Moreover, the clinical phenotype is age-dependent. NMOSD and ON occur more commonly in adults, and ADEM is more common in children [10, 17], which is consistent with the results of the pediatric population studied in our hospital. One of the differences in MOGAD between European children and the children in our study is that ON is more common in Europe (second only to ADEM), whereas in our study, the non-ADEM encephalitis phenotype was more common. This difference may be related to differences in individual anatomical susceptibility to MOG-Abs at different stages in different countries and regions. Children with non-ADEM encephalitis usually experience seizures, and 12% also have meningeal involvement such as headache, nausea, and fever. In our study, 16.7% had seizure symptoms, and up to 10% of patients displayed seizures as the first symptom, which was within the range of the proportion of children with MOGAD who experienced seizures in reports by previous scholars (10.3%–24%). Therefore, for some epileptic seizures that cannot be explained by other means, the possibility of MOGAD should be considered, and MOG-Ab titer detection should be done as soon as possible. A recent study reported that 11.9% of MOG-Ab-positive children also have anti-NMDAR encephalitis [18], and such children are more likely to experience emotional instability, language impairment, and memory loss, among other symptoms; this is consistent with the clinical symptoms of the two children with NMDAR antibodies detected in our study. In addition, no patients with overlap syndrome in our study were found to have anti-AQP-4 antibodies, which may be because NMOSD was relatively rare in our study population (anti-AQP-4 is an important biological marker of NMOSD).

Previous studies [19, 20] have shown that MOG-Ab-positive patients have higher CSF white blood cell counts and protein levels than MOG-Ab-negative patients; however, no obvious difference was found in our study. OCBs can indicate CNS inflammation, but they cannot be used as a marker in MOG-Ab-positive patients. Some scholars have proposed that the detection rate of OCBs in patients with MS can reach 80%, whereas the detection rate in MOG-Ab-positive patients was only 7–11% in one study [21]. Analogously, the detection rate of CSF OCBs in children with MOG-Ab-positive demyelinating CNS disease in our study (10%) was also very similar to the rate reported in that study. Previous studies have suggested that vitamin D supplements play a beneficial role in preventing autoimmune diseases [22]. However, there was no evidence proving that vitamin D levels are related to MOGAD in this study. This may be because of the small sample size.

Most children with MOGAD experience a monophasic course and have a good prognosis. However, with the extension of follow-up times, an increasing number of MOG-Ab-positive patients manifest with multiphasic disease courses [8], such as multiphasic disseminated encephalomyelitis (MDEM), ADEM-ON, and relapsing ON (RON). In recent years, the relationship between MOG-Ab titers and clinical disease activity has remained an active area of research interest. One study found that at the last relapse or follow-up among 10 children with MOGAD, the median MOG-Ab serum titers in relapsed patients were significantly higher than those in non-relapsed patients [23]. Some scholars believe that MOG-Ab titers usually fluctuate dynamically; for example, the titer level can decrease with time in relation to disease remission [20, 24], and it could predict disease activity and recurrence risk [25, 26]. It has also been suggested that patients with persistently positive MOG-Ab titers have a higher probability of disease relapse. Barbagallo et al. proposed that antibody titers in CSF are closely related to the clinical course of autoimmune encephalitis and remain elevated in patients experiencing disease recurrence or in those exhibiting a lack of clinical improvement [27]. Our study also concluded that the level of the MOG-Ab titer may affect the MOG-Ab titer prognosis, and the prognosis of the MOG-Ab titer will further affect disease recurrence. The relapse rate associated with MOG-Ab positivity in our hospital was 33.3%, which is consistent with that of other studies [28, 29]. Therefore, many scholars believe that for children with early demyelination, early detection of MOG-Ab titers and continuous MOG-Ab monitoring and follow-up in the later period have important clinical guiding significance. Thus, a large number of samples is required to verify whether the presence of MOG-Ab can reliably predict relapse and distinguish monophasic from relapsed patients, as well as to further determine whether the titer at relapse is elevated relative to that at remission and whether it can be used for managing children in a clinical setting.

The choice of treatment after the diagnosis of MOGAD is crucial in determining the prognosis of the disease. At present, treatment methods for MOGAD are limited. In the acute phase, hormone shock and IVIG are mostly effective, and immunosuppressive agents should be considered if the initial treatment effect is not optimal [30]. The recurrence of the disease can be prolonged; for example, we found that the time course of the disease course was associated with the time to initial treatment and the MOG-Ab titer prognosis. This may be related to the fact that early aggressive immunotherapy is beneficial in reducing the risk of disease recurrence [31]. However, the specific treatment time and whether the hormone and/or IVIG treatment time can be determined according to the MOG-Ab titer remain controversial. The European children’s expert consensus proposed that the longest time for oral administration of hormones should be 3 months [32]. In the expert consensus for adults, long-term use of hormones for 6–12 months is required. Some scholars have also suggested that most relapses occur when corticosteroids dose is < 10 mg, and the recurrence rate of patients treated with corticosteroids for < 3 months is doubled. Therefore, the reduction in corticosteroid dosage over time needs to be studied further [33, 34]. Previous animal studies [35, 36] reported that interleukin-6 expression continuously increased in recurrent MOG-Ab-positive models, indicating that, in addition to MOG-Abs, exploring other specific biological markers related to disease outcomes could be of immense significance for optimizing the treatment of MOGAD in Chinese children.

Imaging plays a vital role in identifying ADSs in children. Brain MRI manifestations of MOGAD are diverse, although the most common is the ADEM phenotype, which manifests as scattered lesions in the cortex/juxtacortical lesions, with deep nuclei and fiber bundles, as well as high T2 or T2-weighted-fluid-attenuated inversion recovery (FLAIR) signal intensity [25]. The European consensus mentioned that in MOG-Ab-negative children with ADEM, brain MRI may show smaller lesions with clear boundaries, or it may only reveal the involvement of some anatomical areas [37]. The brain MRI of MOG-Ab-positive children with ADEM shows larger, bilateral, and extensive lesions, and the clinical results are better when compared with those of MOG-Ab-negative children [2]. Studies have also suggested that regardless of whether a patient is MOG-Ab positive or negative, those with ADEM have similar imaging characteristics. Children with ADEM were not assessed separately in this study, and when compared with MOG-Ab-negative children, the brain MRI lesions of MOG-Ab-positive children were more likely to exhibit the involvement of juxtacortical lesions and lesions in the temporal lobe. In addition, the lesions exhibited unique shapes; this may have been related to the higher incidence of MOG-related cortical encephalitis in our group, and MOG, as a glycoprotein of oligodendrocytes, is associated with pathological features located in the outermost myelin sheath [38]. Furthermore, during follow-up, it was found that the time for improvement of brain MRI in the MOG-Ab-positive children was relatively longer than that in the MOG-Ab-negative children. This is one of the reasons why MOG-Ab-positive children are more likely to experience relapse. A previous study found that in the spinal cord MRI of MOG-Ab-positive children, the cervical segment was more involved than the thoracic segment [19], which is consistent with our observations. However, in contrast to that study, long-segment involvement of the spinal cord in our study was uncommon, which may have been owing to our small sample size and the low popularity of spinal cord MRI. Unfortunately, we were unable to perform orbital MRI and optic nerve MRI for relevant statistical analyses because of the relatively small number of children with ON.

In summary, the clinical and MRI manifestations of MOGAD in children are diverse, and the clinical disease spectrum is still expanding, accounting for an increasing proportion of neurological and immunological diseases. Since this was a retrospective study, the small sample size and short follow-up time are among its limitations. In future research, multi-center sample populations should be studied, the follow-up time should be extended, and a prospective controlled study should be conducted to further explore and compare the clinical characteristics of MOGAD in Chinese children. This will improve the general understanding of MOGAD, reducing the number of misdiagnoses of the disease among children. In addition to the existing treatment plan, an appropriate diagnosis and treatment plan should be developed to reduce recurrence and improve the prognosis of children with MOGAD.

### Data availability statement

The original contributions presented in the study are included in the article; further inquiries can be directed to the corresponding authors.

## Abbreviations

ADEM: Acute disseminated encephalomyelitis
ADS: Acquired demyelinating syndrome
AQP4: Aquaporin-4
CASPR2: Contactin-associated protein-like 2
CBA: Cell-based assay
CIS: Clinically isolated syndrome
CNS: Central nervous system
EU: European Union
FLAIR: Fluid-attenuated inversion recovery
IgG: Immunoglobulin G
IVIG: Intravenous immunoglobulin
HEK293: Human embryonic kidney 293 cells
MOG: Myelin oligodendrocyte glycoprotein
MOG-Ab: Myelin oligodendrocyte glycoprotein antibody
MOGAD: Myelin oligodendrocyte glycoprotein antibody-associated disease
MRI: Magnetic resonance imaging
MS: Multiple sclerosis
MDEM: Multiphasic disseminated encephalomyelitis
NMDAR: N-methyl-D-aspartate receptor
NMOSD: Neuromyelitis optica spectrum disorder
OCB: The oligoclonal-IgG band
ON: Optic neuritis
pcDNA3.1-EGFP: Plasmid cloning deoxyribonucleic acid 3.1-enhanced green fluorescent protein
RON: Relapsing ON
TM: Transverse myelitis
WBC: White blood cell
